# Immediate Effects of Nasalance Exercises on Patients with Organic Dysphonia

**DOI:** 10.1177/19160216251333360

**Published:** 2025-05-21

**Authors:** Liudmila Kuranova, Marie-Anne Kainz, Matthias Echternach, Michael Döllinger, Marie Köberlein

**Affiliations:** 1 Division of Phoniatrics and Pediatric Audiology, Department of Otorhinolaryngology, University Hospital LMU Munich, Bavaria, Germany; 2 Division of Phoniatrics and Pediatric Audiology, Department of Otorhinolaryngology Head & Neck Surgery, University Hospital Erlangen, Friedrich-Alexander-University Erlangen-Nürnberg, Erlangen, Bavaria, Germany

**Keywords:** vocal fold mass lesion, organic dysphonia, nasalance exercises, high-speed videoendoscopy, voice therapy, resonant voice

## Abstract

**Importance:**

Nasalance exercises (also known as resonance exercises) are widely used in voice therapy. Understanding their effects can guide therapeutic approaches and surgical decisions.

**Objective:**

To analyze the immediate effects of nasalance exercises on vocal fold oscillation in patients with vocal fold mass lesions and a recommendation for phonosurgery.

**Design:**

Prospective observational study following the STROBE guidelines.

**Setting:**

Department of Phoniatrics, university hospital.

**Participants:**

Seven patients with vocal fold mass lesions (6 with polyps, 1 with Reinke edema) and indication for surgery.

**Intervention/Exposures:**

Participants performed nasalance exercises for 10 minutes. Recordings were taken before the exercise (pre), immediately after (post0), and 10 minutes after completion (post10). Subjects phonated vowel [i:] on a sustained pitch (250 Hz for females, 125 Hz for males) at a comfortable level of loudness.

**Main Outcome Measures:**

Data were collected using transnasal high-speed videoendoscopy, a Rothenberg mask for airflow measurement, electroglottography, and audio recordings. Extracted parameters were: nasalance, open quotient (OQ), closing quotient (ClQ), sound pressure level (SPL), Jitter, and cepstral peak prominence (CPP).

**Results:**

Nasalance increased immediately after the exercises for 6 out of 7 subjects. OQ values varied: they increased in 3 subjects, decreased in 3, and remained unchanged in 1. No consistent relationship was found between SPL and ClQ. Jitter increased in 5 subjects. CPP did not show clear tendencies. The effects on voice parameters did not persist 10 minutes postexercise. There were no significant correlations with age, sex, or preintervention voice indices (Voice Handicap Index, Dysphonia Severity Index).

**Conclusions:**

In patients with organic dysphonia and an indication for surgery, a raised nasalance value directly after the execution of nasalance exercises does not necessarily lead to stabilized voice parameters, and the possible effects do not seem to be persistent.

**Relevance:**

Nasalance exercises might not provide sustained benefits in stabilizing vocal fold vibrations in subjects with an indication for surgery.

## Graphical abstract

**Graphical Abstract:**
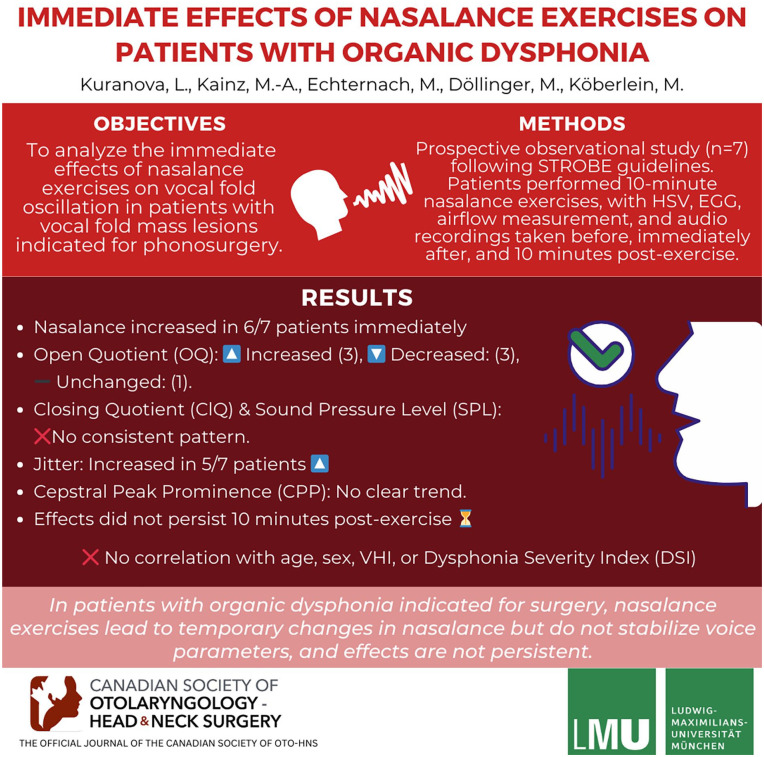

## Introduction

Dysphonia is frequently caused by vocal fold mass lesions such as polyps, Reinke edemas, cysts, and nodes.^1-4^ Due to the stiffness and volume effects that come with the mass lesions, the movement of the vocal folds during oscillation is impaired, producing aperiodicities, asymmetries, phase differences, or incomplete closure of the glottis.^5^ As a consequence, the transglottic airflow is distorted resulting in dysphonia. The main symptoms are described as hoarseness, reduced volume of voice, vocal fatigue, low pitches, and dyspnea.^1^ Further symptoms could be instabilities in especially demanding phonation activities, such as register transitions in professional voice users.^6^ The treatment of dysphonia due to benign vocal fold mass lesions can be performed with pharmacotherapy, voice therapy, or phonomicrosurgery. The goal of all the therapy approaches is the stabilization of vocal function, which is related to the improvement of oscillation of the vocal folds or the entrainment of their oscillatory eigenmodes.^7,8^ For this purpose, nasalance excercizes such as phonation on [m], [n] and [ng] are frequently used in voice therapy.^5,9-11^ These types of exercises are also known as resonance exercises, voice placement, resonant voice, or humming. First introduced by Fletcher and Frost in 1974, the term “nasalance” has been described as a measure of velopharyngeal closure during phonation by comparing nasally emitted acoustic energy to orally emitted energy.^12^ Using nasalance exercises, a raise of the nasalance value is expected due to the lowering of the soft palate and a bigger velopharyngeal port opening. The nasal tract is linked to the oral and pharyngeal parts of the vocal tract, and the impedance of the entire vocal tract is being increased primarily because of the added complexity of the sound path, the narrower cross-sectional area of the nasal passages, and the absorptive properties of the nasal mucosa. This can facilitate vocal fold oscillation at an improved reactance level.^13^ Therefore, nasalance exercises aim to stabilize voice production.^14-16^ Stabilized vocal fold oscillation might show higher sound pressure level (SPL) due to a greater vocal fold closure and/or smaller closing quotient (ClQ), that is, a faster closing phase and therefore a stronger molecular compression.^17-21^ Additional positive effects of nasalance exercises regarding functional aspects are the release of supraglottic compression^22^ and a reduced adduction before phonation onset.^23^ In patients with organic dysphonia, Vlot et al showed better perturbation parameters in humming compared to natural sustained phonation on the vowel [e:].^24^ In studies by Dursun et al^25^ and Cho et al,^26^ it was shown that the size of polyps was associated with acoustic perturbation parameters reflecting vocal quality.

In principle, an optimized increase of nasalance could be used for stabilization of voice in instable vocal zones, such as register transitions in singing.^27^ An increased nasality can also result in changes of the resonatory properties.^28,29^ It has been shown that the opening of the velopharyngeal port influences the first resonance frequency^30^ as well as the radiated voice spectrum.^31^ Additionally, nasality is required as a part of normal speech for several languages.^32^ However, nasalance that is too high, that is, the opening of the velopharyngeal port is too large, due to dysfunction or incompetence, can result in unwanted acoustical effects, resulting in a rhinophonia aperta/hypernasality.

Although it has been reported that voice therapy can show positive effects in patients with organic dysphonia,^9,24^ evidence that (1) there are immediate effects from nasalance exercises on the phonation mechanism, and (2) this could be advantageous in patients with an indication for surgery, that is, with larger vocal fold lesions, is lacking.

## Material and Methods

**Figure 1.**
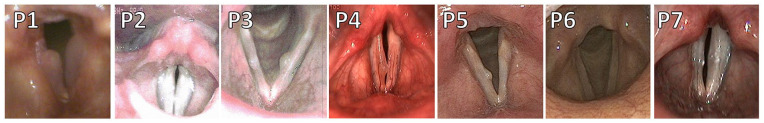
The subjects’ vocal fold mass lesions.

After the approval of the local ethical committee and obtaining informed written consent from all participants, 8 subjects with diagnosed vocal fold lesions (Table 1 and Figure 1) were asked to execute a 10-minute nasalance exercise protocol, instructed and accompanied by a speech-language therapist from the ENT Department. The protocol is given in Table 2. The subjects were recorded at 3 time points: before the exercises (pre), directly after (post0), and 10 minutes after (post10) the exercises. For the measurement, they phonated on a sustained vowel [i:] at a comfortable loudness level. In order to avoid strain, the fundamental frequencies of 250 Hz for females and 125 Hz for males were chosen, for they lie in the most comfortable range for the speaking voice and sustained phonation. The subjects got their respective pitch played on a piano. The recordings were conducted with a high-speed video-camera (20,000 fps, Fastcam SA-X2; Photron, Tokyo, Japan) using transnasal endoscopy (ENF GP, Fa.; Olympus, Hamburg, Germany) through a Rothenberg mask (divided mask with PT-2E Transducer; Glottal Enterprises, Syracuse, NY, USA) as described previously.^27^ Audio signals (IMK SC 4061 microphone, DPA microphones, Alleroed, Denmark or Sennheiser ME 62; Sennheiser, Wedemark, Germany) and electroglottographic (EGG) signals (EG2-PCX2; Glottal Enterprises) were recorded simultaneously. For the calibration of the audio-signal, a sound level meter was used (Voltcraft 322, Hong Kong, China) and for the flow the FC-1 and PC-1 calibrators (Glottal Enterprises). The data of 1 subject had to be excluded from further analysis due to a signal interference.

**Table 1.** The Subjects’ Sex, Age, Pathology, Lateralization, Initial Dysphonia Severity Index and Voice Handicap Index.

Subject	Sex	Age	Pathology	Pathology’s lateralization	DSI	VHI
P1	F	61	Reinke’s edema	Both sides	1.0	22
P2	F	29	Polyp	Both sides	4.4	2.2
P3	M	35	Polyp	Left	−1.2	25
P4	F	41	Polyp	Right	1.8	29
P5	F	54	Polyp	Right	3.2	49
P6	M	33	Polyp	Left	0.14	49
P7	F	39	Polyp	Both sides	3.6	20

Abbreviations: DSI, Dysphonia Severity Index; VHI, Voice Handicap Index.

**Table 2.** The Nasalance Exercise Protocol Used in This Study.

Duration (minutes)	Task
2	Alternating pitch on /m/ within the musical interval of a major second
1	Rest
2	Glissandi on /m/, /n/, /ng/ within the musical interval of a fifth
1	Rest
2	Alternating pitch on /mom/, /non/, /ngong/ within a major second
1	Rest
2	Alternating pitches on /m/ within a major second

The high-speed images were preprocessed as described earlier.^27^ Afterward, the glottis was segmented using the software Glottis Analysis Tools.^33,34^ The software Sopran (Svante Granqvist, Karolinska Insititute, Stockholm, Sweden) was used to calibrate the audio and flow signals. The proportional nasalance value was calculated by dividing the nasal flow by the total flow from added nasal and oral flow signals. All signals were brought together in the software Multi Signal Analyzer (Division for Phoniatrics, FAU Erlangen-Nuremberg, Germany), where the mean values of the parameters listed in Table 3 were calculated for a window of 500 ms. Due to the small number of subjects, statistical analysis was not considered meaningful.

**Table 3.** Source, Signals, and Resulting Parameters.

Source	HSV	EGG	Microphones, sound level meter	Rothenberg-mask
	⇩	⇩	⇩	⇩
Signals	GAW	EGG waveform	Audio tracks	• Oral flow • Nasal flow
	⇩	⇩	⇩	⇩
Resulting parameters	• OQ • ClQ • CPP	• Fundamental frequency *f*_o_ • CPP	• SPL • Jitter • CPP	Nasalance

Abbreviations: ClQ, closing quotient; CPP, cepstral peak prominence; EGG, electroglottography; GAW, glottal area waveform; HSV, high speed video; OQ, open quotient; SPL, sound pressure level.

## Results

**Figure 2.**
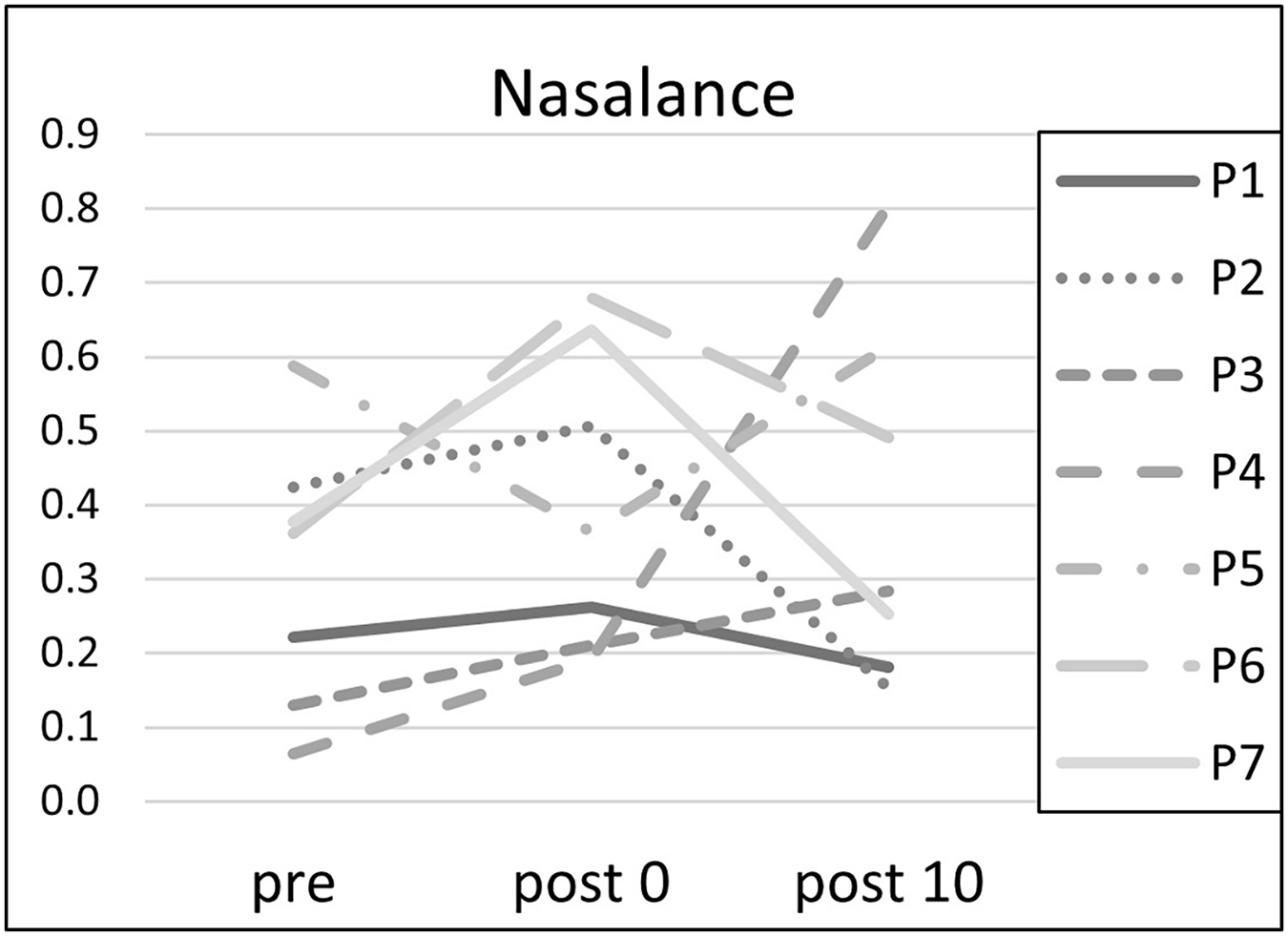
Nasalance at the points of measurement (pre = before the exercises, post0 = after nasalance exercises and post10 = 10 minutes after the exercises).

The nasalance was raised in 6 of 7 subjects directly after the exercises (Figure 2). There was no uniform accompanying effect concerning the other parameters.

**Figure 3.**
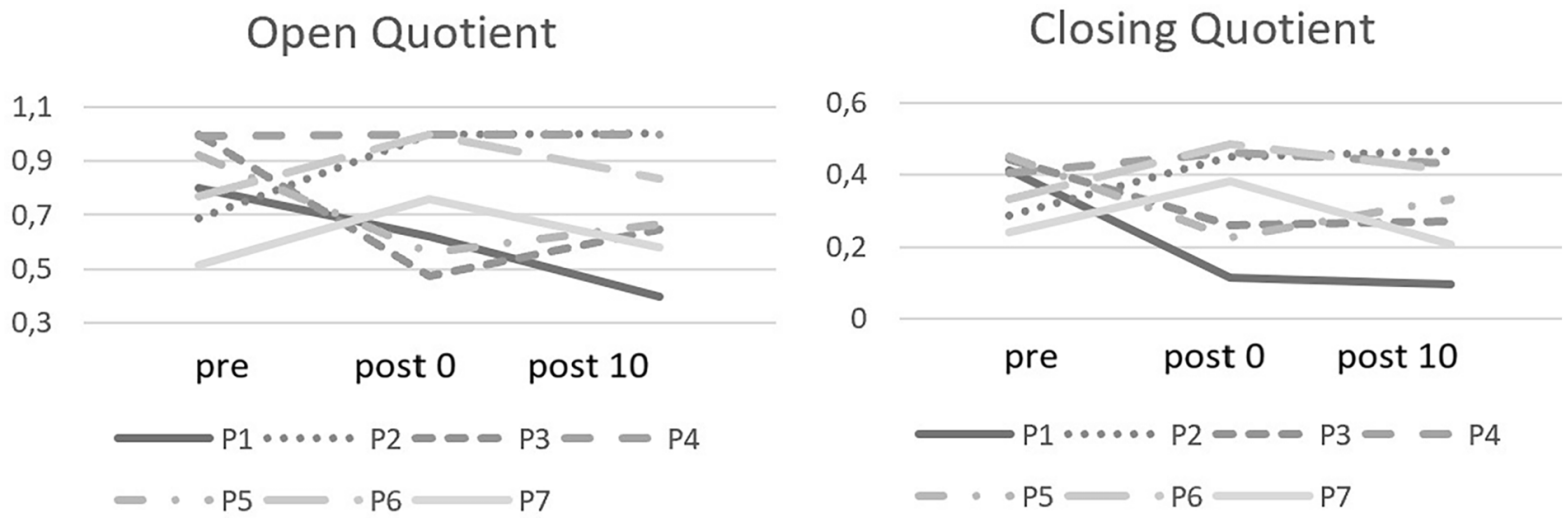
OQ and ClQ from the GAW for the points of measurement (pre = before the exercises, post0 = after nasalance exercises and post10 = 10 minutes after the exercises). ClQ, closing quotient; GAW, glottal area waveform; OQ, open quotient.

Directly after the exercises, the open quotient (OQ) glottal area waveform (GAW) showed higher values in 3 cases (P2, P6, P7), lower values in 3 cases (P1, P3, P5), and remained almost unchanged in subject P4 (Figure 3). The ClQ showed a corresponding behavior to OQ GAW: directly after the exercises, it was lowered for P1, P3, P5, and raised for the other subjects, with P4 exhibiting the smallest difference. Ten minutes after the exercises, the OQ values moved back toward the prevalues except for P1, where the value had declined further, and P2, where the value stayed at the post0-level. At timepoint post10, the ClQ values of P1, P2, and P3 remained approximately at the post0-level.

**Figure 4.**
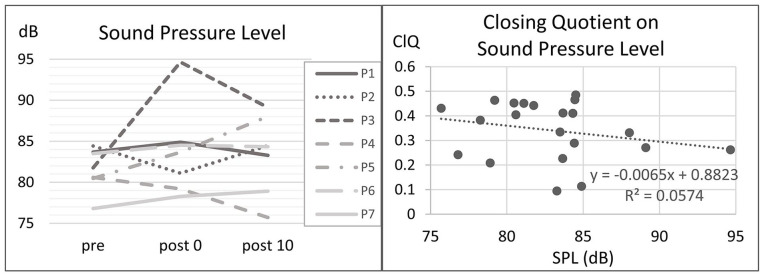
Left: SPL for all subjects with respect to the points of measurement (pre = before the exercises, post0 = after nasalance exercises and post10 = 10 minutes after the exercises). Right: Closing quotient plotted against SPL. SPL, sound pressure level.

SPL was lowered directly after the exercises for P2 and P4 and raised for the others with P3 as outlier with an increase of 12,9 dB (Figure 4, left). There was no relation between SPL and ClQ (Figure 4, right).

**Figure 5.**
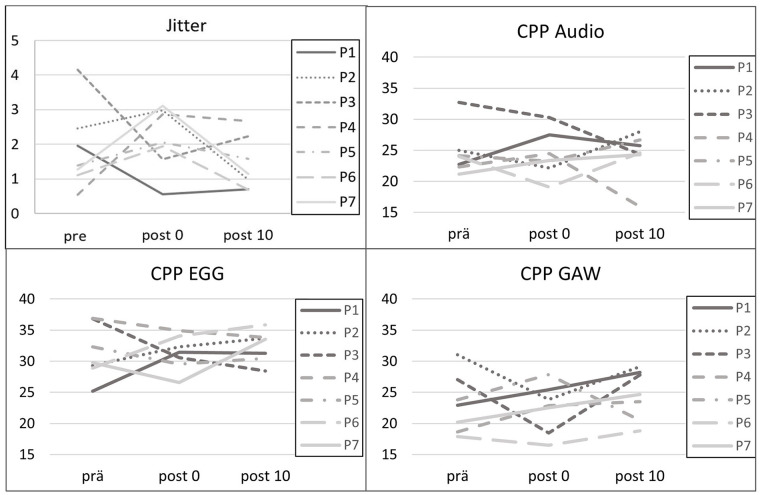
Jitter from the audio signal and CPP of audio, EGG and GAW with respect to the points of measurement (pre = before the exercises, post0 = after nasalance exercises and post10 = 10 minutes after the exercises). CPP, cepstral peak prominence; EGG, electroglottography; GAW, glottal area waveform.

The jitter increased in 5 of 7 subjects directly after the exercises (Figure 5). Subject P5, who was the only one with decreased nasalance post0, showed a decreased OQ GAW and a slightly raised jitter. The jitter was lowered for P1 and P3 directly after the exercises. Ten minutes after the exercises, the values had moved back in line with the pre-level for all of the participants. Post10 values were lower than the prevalues for P1, P2, P3, and P6. The cepstral peak prominence (CPP) values for audio, EGG, and GAW did not show clear tendencies (Figure 5).

**Figure 6.**
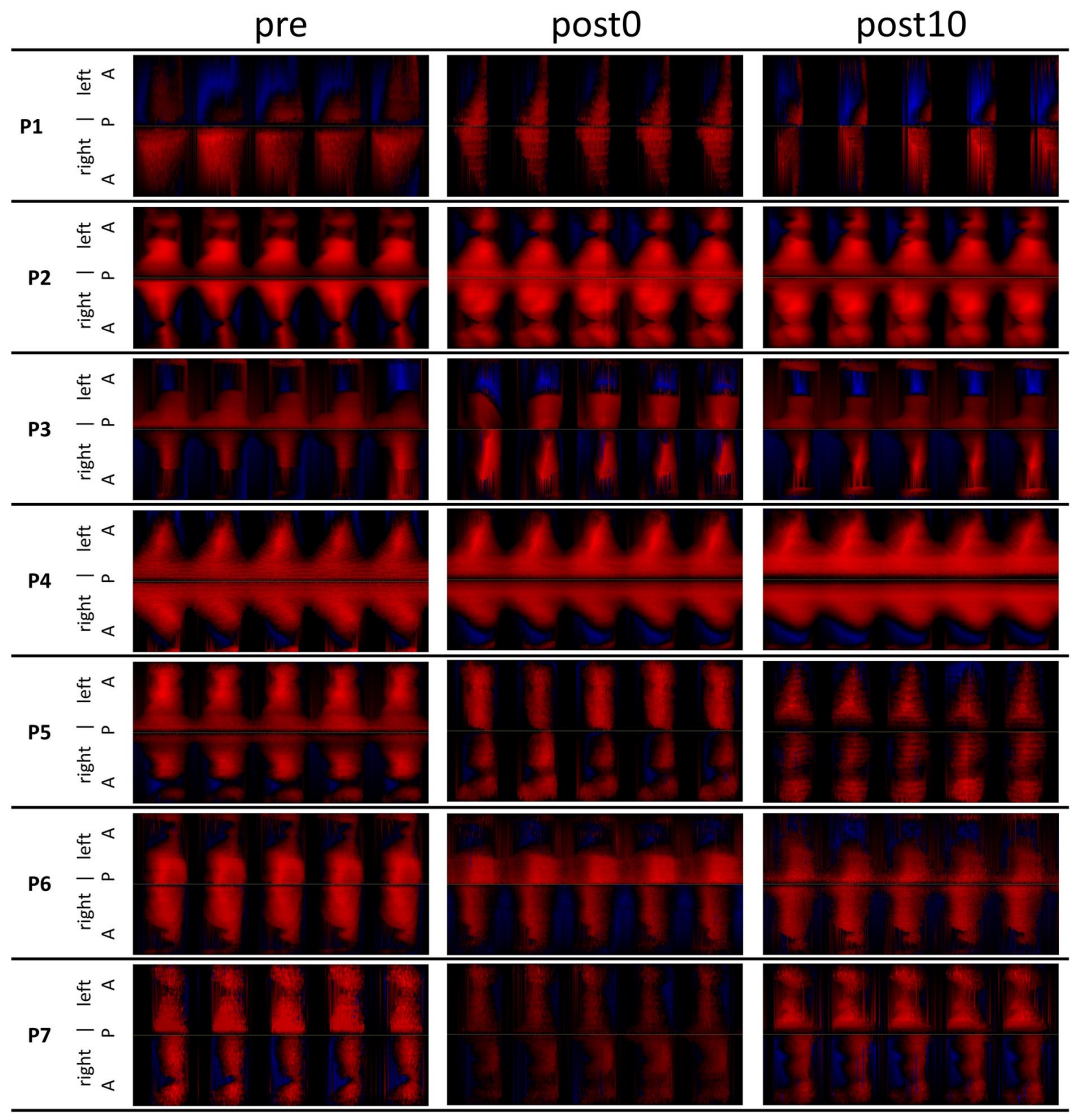
PVG of 5 oscillation cycles for each subject performing a vowel [i:] before, immediately after, and 10 minutes after nasalance exercises. PVG, phonovibrograms.

No dependency of the parameters regarding age, sex, type of diagnosis, Voice Handicap Index (VHI), and Dysphonia Severity Index (DSI) was found. Phonovibrograms do not show any visually observable events (Figure 6).

## Discussion

Possibilities for the treatment of benign vocal fold mass lesions are situated in the fields of pharmacotherapy, voice therapy, and phonomicrosurgery.^1^ This study analyzed the immediate effects of nasalance exercises in 7 subjects with vocal fold mass lesions and indications for surgery. Even though the nasalance was raised directly after the exercises, suggesting an impact from the treatment, the investigated parameters showed heterogeneous behavior. Also, the alterations of the parameters observed immediately after the exercises were not persisting 10 minutes after the exercises.

Nasalance exercises can help principally to stabilize or facilitate phonation, and they find application in many different areas; for example, in speech language therapy for malregulative voice disorders, in voice pedagogy, and with professional voice users. Contrary to the expectation that nasalance exercises could improve voice perturbation measures also in patients with organic dysphonia,^24^ in this study, the GAW-based OQ, as a possible sign for voice stability, did not develop in a direction corresponding to the increase in nasalance. Also, the increased Jitter values right after the exercises do not fulfill the expectation of stabilized voice after nasalance exercises, and they rather indicate a worsening of the voice quality. Since jitter is considered problematic for the detection of a vocal outcome after treatment, the CPP values were also added in order to account for potentially non-quasi-periodic signals.^35,36^ However, also CPP showed no clear tendencies. Observations of deterioration after vocal exercises had already been made in a study on immediate effects of water resistance therapy (WRT), which is also a common voice therapy method that uses similar principles to nasalance exercises.^37^ In healthy voices or malregulative voices, nasalance increases the impedance of the vocal tract and optimizes the pressure difference at the bottleneck of the glottis, which facilitates the mucosal wave. Due to the organic pathologies, this effect might not be possible. The tissues of the lamina propria might be more immobile and under tension because of the thickening. A thickening might, furthermore, interfere with the physical principles of self-sustained oscillation: It might prohibit the desired opening of the glottis as well as the full closure. The inconsistency of the results might be founded in different subtypes of vocal fold mass lesions, or the personal compensation strategies of the subjects, which can also be connected to the cause of the vocal fold mass lesions, that is, possible functional disorders in voice use.^38^ The underlying triggers for the mass lesions of the investigated subjects of this study are unknown. However, no connection between DSI, VHI, or lateralization could be found. In order to identify such subtypes of mass lesion and their reaction to nasalance exercises, the investigation of larger cohorts will be necessary. In addition, it could be useful to identify the relative size and position of the thickening and the resulting oscillation patterns. Such an approach has been made by Martinez-Paredes et al.,^39^ who investigated unilateral mass lesions regarding their size, laterality, and location. However, no systematic links to voice quality were found.

In the presented dataset, no correlation between SPL and ClQ or other perturbation measures was found. In another study on vocal fold oscillation patterns related to loudness in patients with vocal fold mass lesions, changes of voice parameters were shown for different SPL levels. However, a direct correlation could not be proven.^20^ An explanation for the outcome in the presented study might be the general and profound system disruption of the investigated voices.

Vlot et al presented improved voice parameters during a humming phonation in patients with organic dysphonia.^24^ It is possible that the positive effects occur during the treatment but cannot be preserved in usual phonation, that is, speaking voice with open mouth and no emphasis on nasal connection. Such had also been observed in the earlier mentioned study on WRT regarding patients with vocal fold mass lesions.^37^

It is questionable if a single 10-minute session of nasalance exercises is enough to show direct effects for larger vocal fold mass lesions. Since organic dysphonia is frequently linked to functional disorders, perhaps the patients would need long-term voice therapy to exhibit the effects of a generally healthier voice use, as has already been considered by Ogawa and colleagues^23^ and Vlot et al.^24^ Even though the effects of the nasalance exercises were not persistent, the jitter, 10 minutes after the exercises, was lower than before the exercises in 4 of the 7 subjects. This might be a hint of a possible long-term improvement. It might be hypothesized that the nasalance exercise puts stress on the voice system, since the subjects have to phonate against increased resistance. If the resistance is lowered again, the system might show an aftereffect of the increased stress at first, but untighten and improve after a few minutes. Furthermore, it might be the case that the chosen protocol did not offer the right exercises for the particular patients, and that large mass lesions might require different protocols.

## Limitations

The indication for surgery had already been given for the investigated subjects. This means they already exhibited strong organic changes. In subjects with mass lesions of smaller extents or different types, positive effects from nasalance exercises might be achievable.^1,5,11^ The cohort of this study was rather small due to the complexity of the experimental setup, and also because some potential subjects preferred to undergo surgery directly. This is also the reason why the presented study could not provide a control group. Furthermore, this study could not provide a completely homogenous or heterogenous cohort, but included 6 subjects with polyps and 1 with Reinke’s edema. Other types of vocal fold mass lesions which would be of interest, such as cysts and nodules, could not be investigated due to a lack of patients in the period of the study.

Another limitation is the transnasal endoscopy which has an effect on the nasalance value itself, since the device runs through the nose and the velopharyngeal port. The effects on vocal tract impedance or the obstruction at the soft palate can, however, be considered systematic errors. Additionally, the subjects could have been irritated by the sensation of the transnasal endoscopy and therefore have shown different behaviors than otherwise might have been the case.

Finally, the DSI was only assessed in the beginning of the study to classify the voice problem. Following DSI measurements, other multiparametric approaches such as AVQI and perceptual voice assessments could have added further insights.

## Conclusions

In patients with organic dysphonia and an indication for surgery, a raised nasalance value directly after the execution of nasalance exercises does not necessarily lead to stabilized voice parameters after the exercises, and the possible effects do not seem to be reliably persistent.
